# Supplementary material for: Targeting enhancer switching overcomes non-genetic drug resistance in acute myeloid leukaemia
Source: Nat Commun. 2019 Jun 20;10:2723. doi: 10.1038/s41467-019-10652-9 (PMC6586637; doi:10.1038/s41467-019-10652-9)

SUPPLEMENTARY INFORMATION:

Targeting enhancer switching overcomes non-genetic drug resistance in acute myeloid leukaemia

Bell, Fennell et al.

- Supplementary Figures and Supplementary figure legends
- Supplementary Note
- Uncropped western blots

Supplementary Figure 1

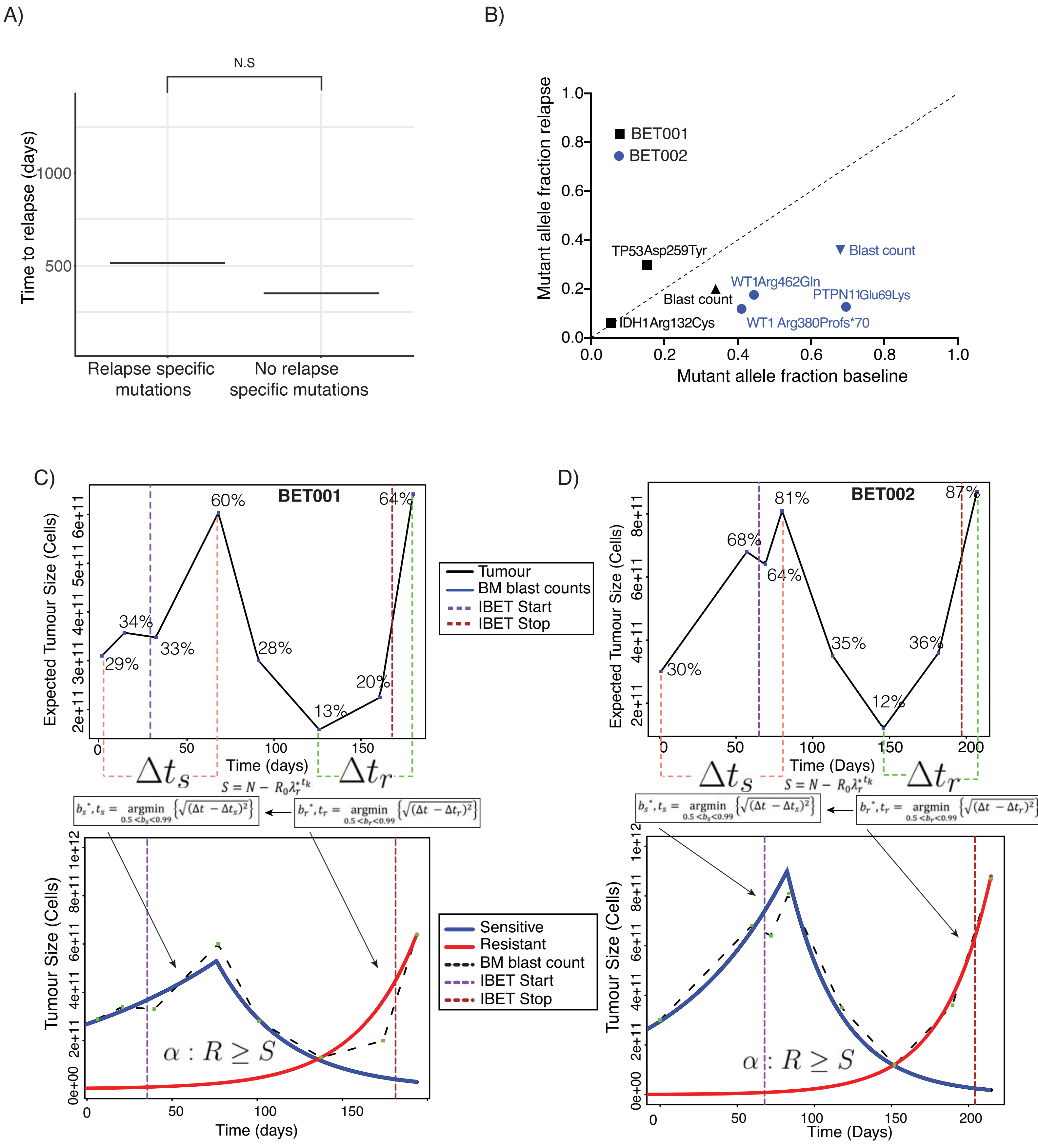

Supplementary Figure 1: **Characterization of non-genomic resistance in patient AML**

(A) Analysis of the time to relapse in AML patients from Li et al. dataset with and without relapse specific non-synonymous mutations in AML genes. N.S = not significant. **(B)** Analysis of mutant allele fraction of known AML mutations using a validated targeted sequencing panel[49] in AML blasts isolated from BET001 and BET002 at diagnosis and relapse. No new mutations were detected in either patient at relapse. **(C)** Mathematical modelling of the growth rate of sensitive and resistant populations with and without drug pressure, based on blast counts for patient BET001. **(D)** Mathematical modelling of the growth rate of sensitive and resistant populations with and without drug pressure, based on blast counts for patient BET002 (detailed description of the analysis and conclusions are provided in Methods and Supplementary Note 1).

Supplementary Figure 2

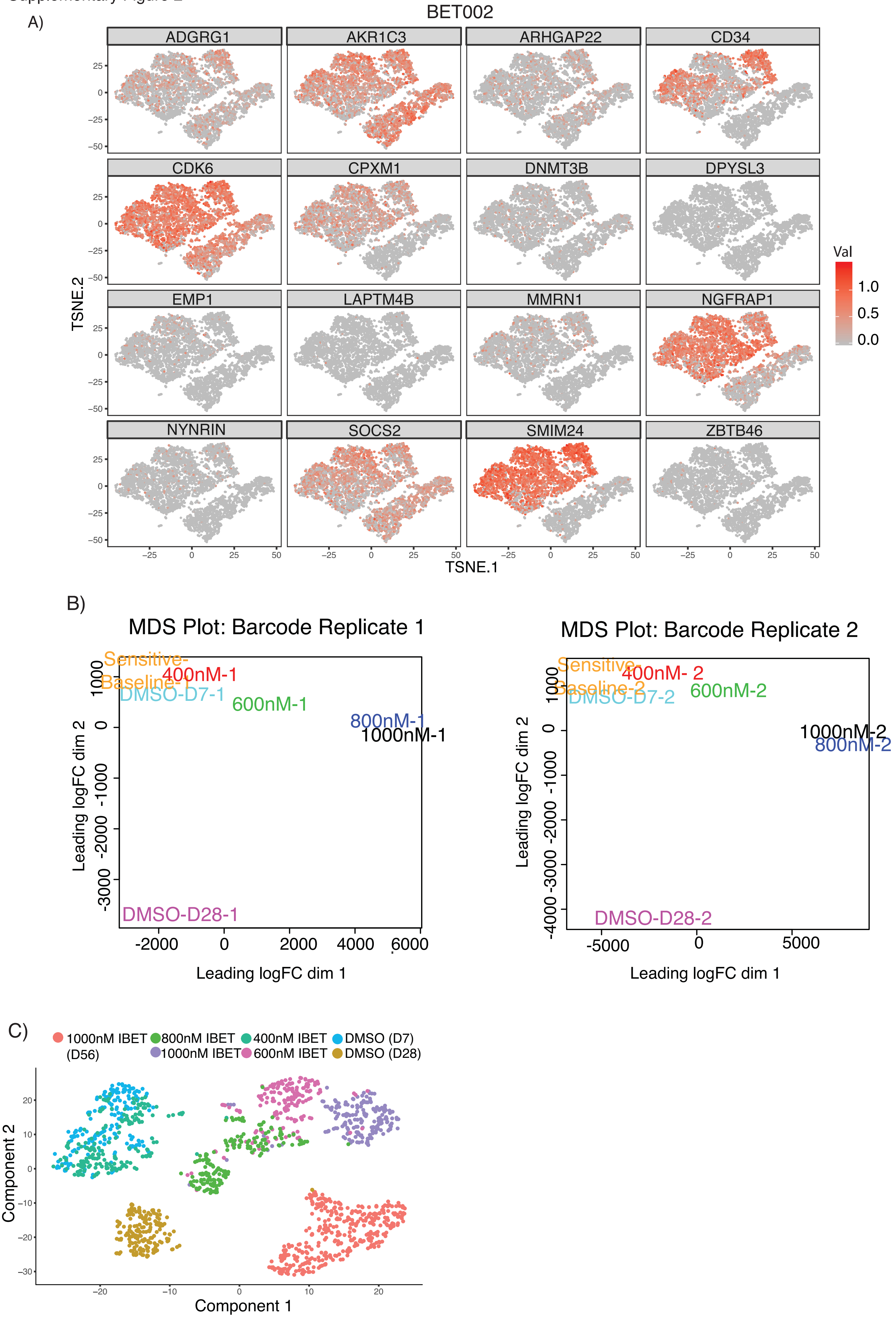

Supplementary Figure 2: **Enrichment of LSC signature at clinical relapse**

(A) Expression analysis of all LSC signature genes (defined in REF [14]) in blast cells from patient BET002 overlaid onto the t-SNE plot. FAM30A (KIAA0125) wasn't detected in any of the leukemic blasts for patient BET002 and therefore is not presented. **(B)** MDS plots clustered by barcode representation for each of the biological replicates from the barcoding experiment shown in Figure 1E. **(C)** t-SNE analysis on a total of 1471 single drug naïve cells treated with either DMSO or increasing concentrations of IBET over a period of 56 days, until the cells became resistant. Analysis was conducted based on differentially expressed genes between resistant versus drug naïve cells from bulk RNA-sequencing.

Supplementary Figure 3

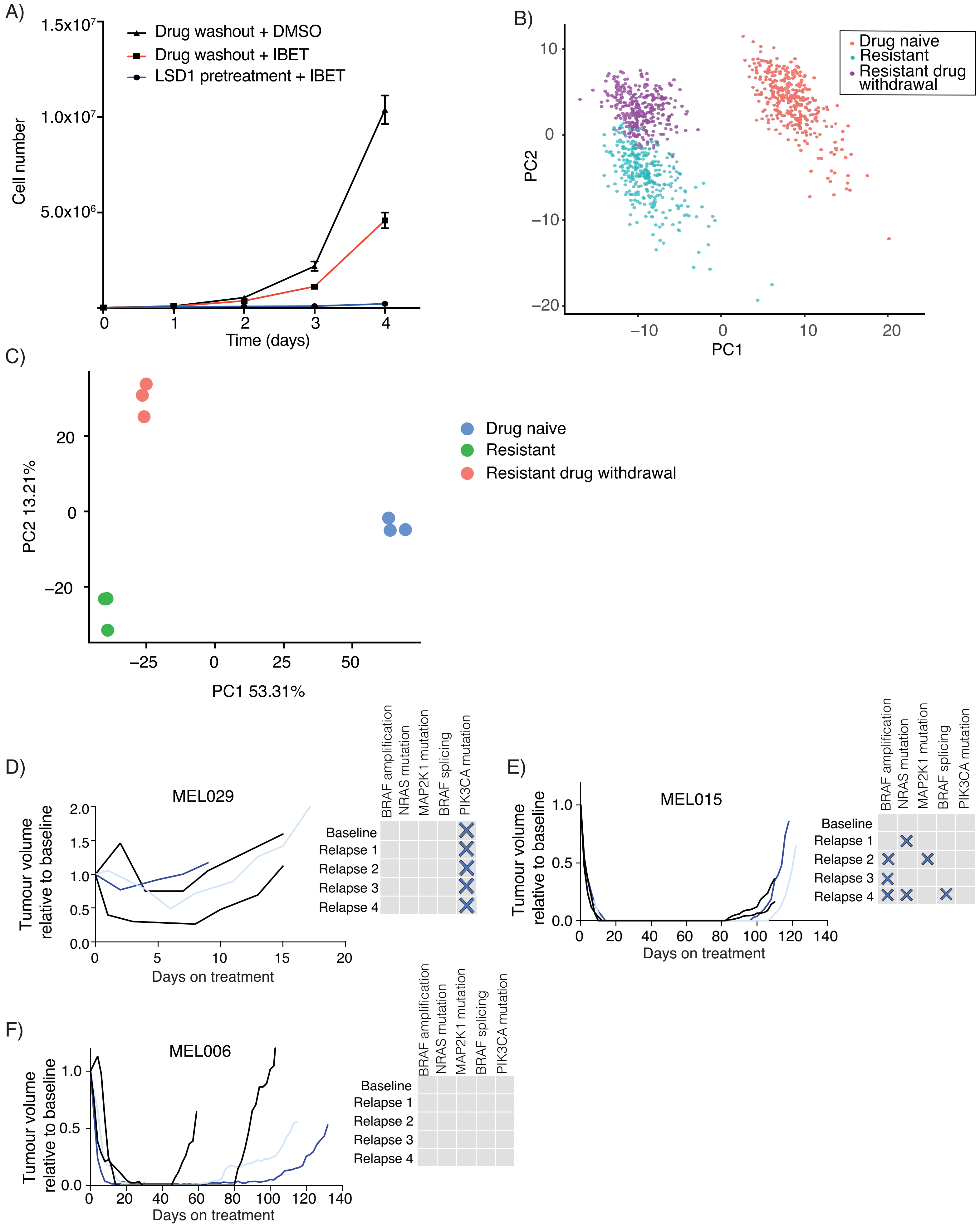

Supplementary Figure 3: Non-genetic resistance can be stable and is not confined to AML or epigenetic therapies

(A) Proliferation assay of resistant cells that have been grown without IBET (drug washout) for 6 days followed by treatment with DMSO or re-exposure to IBET (1000nM). Also shown here is pre-treatment of the resistant cells for 6 days with GSK-LSD1 (500nM) followed by re-challenge with IBET (1000nM). Error bars represent S.E.M of 3 cell culture replicates. Representative of 3 biological replicates. **(B)** Principal component analysis of scRNA-seq data of 979 cells from drug naïve, resistant and resistant 4-day IBET withdrawal cells. **(C)** Principal component analysis of bulk RNA-seq of drug naïve cells, resistant cells and resistant cells withdrawn from IBET (drug washout) for 6 days. **(D-F)** Growth kinetics and mutational status at baseline and relapse of BRAF mutant PDX samples treated with a combination of BRAF and MEK inhibitors. **(D)** MEL029 is a representative example of primary resistance. **(E)** MEL015 is a representative example of acquired genetic resistance. **(F)** MEL006 is a representative example of non-genomic resistance. n = 4 mice for each tumour sample.

Supplementary Figure 4

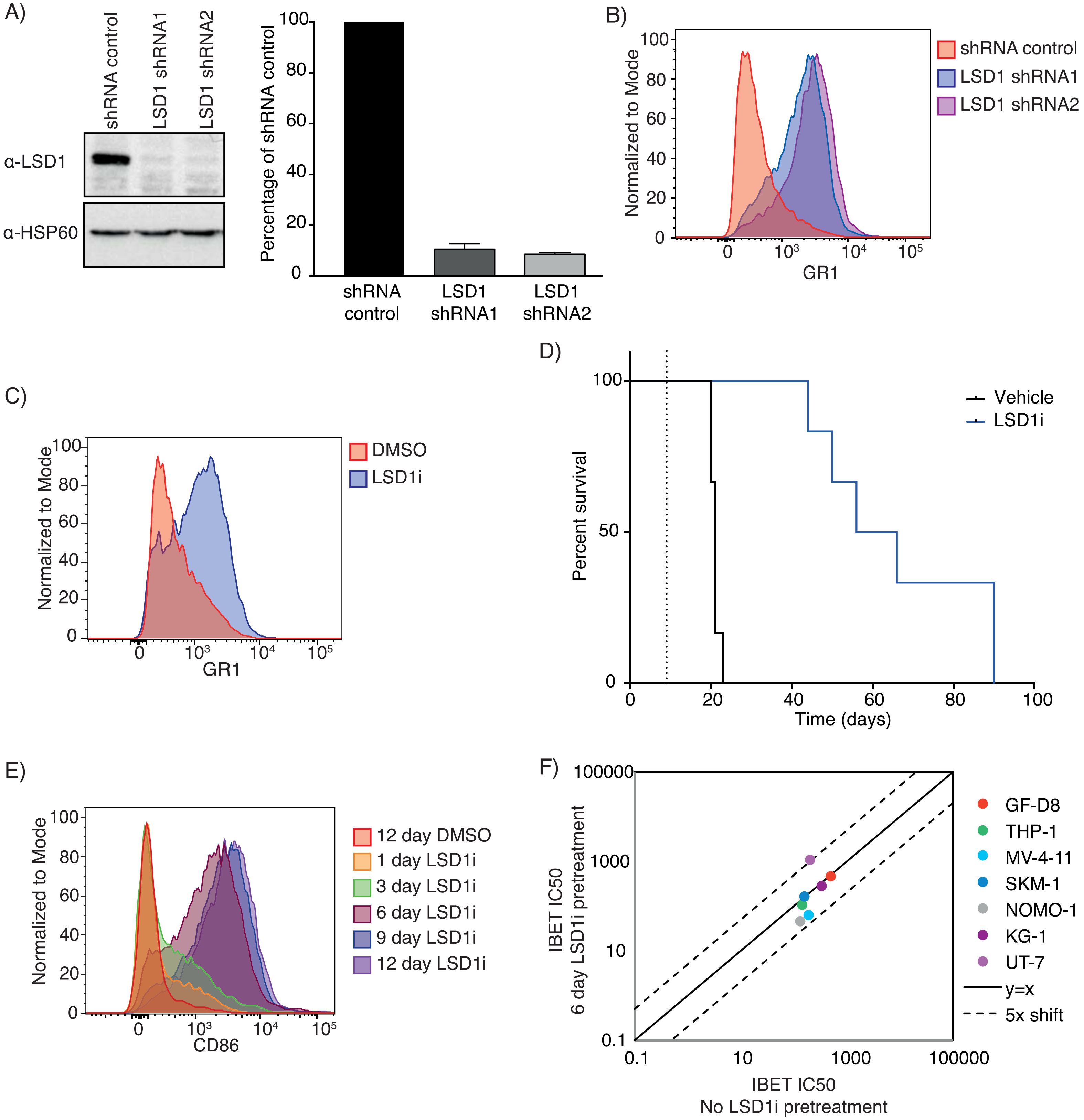

Supplementary Figure 4: **LSD1i overcomes the stable resistance phenotype**

(A) Western blot and qPCR analysis of LSD1 levels in shRNA control and LSD1 shRNA resistant cells. qPCR is from 3 biological replicates. **(B)** Flow cytometry of GR1 expression in shRNA_control and shRNA_LSD1 resistant cells. **(C)** Flow cytometry of GR1 expression in resistant cells treated for 6 days with DMSO or GSK-LSD1i (500nM). **(D)** Kaplan-Meier curve of vehicle and drug treated mice transplanted with MLL-AF9 leukaemic cells serially re-transplanted (4 generations) in the presence of DMSO treatment. n = 6 mice per group. Dotted line indicates the start of treatment. Dosing was performed by IP injection once a day at 0.5mg/kg for GSK-LSD1. **(E)** FACS analyses of CD86 surface expression after dosing of drug resistant cells for different time periods of GSK-LSD1i (500nM). **(F)** Dose response assay (IC50) to IBET at 72hrs of a panel of AML cell lines with or without pre-treatment with GSK-LSD1i (100nM) for 6 days. Dotted lines indicate a 5-fold shift in IBET sensitivity.

Supplementary Figure 5

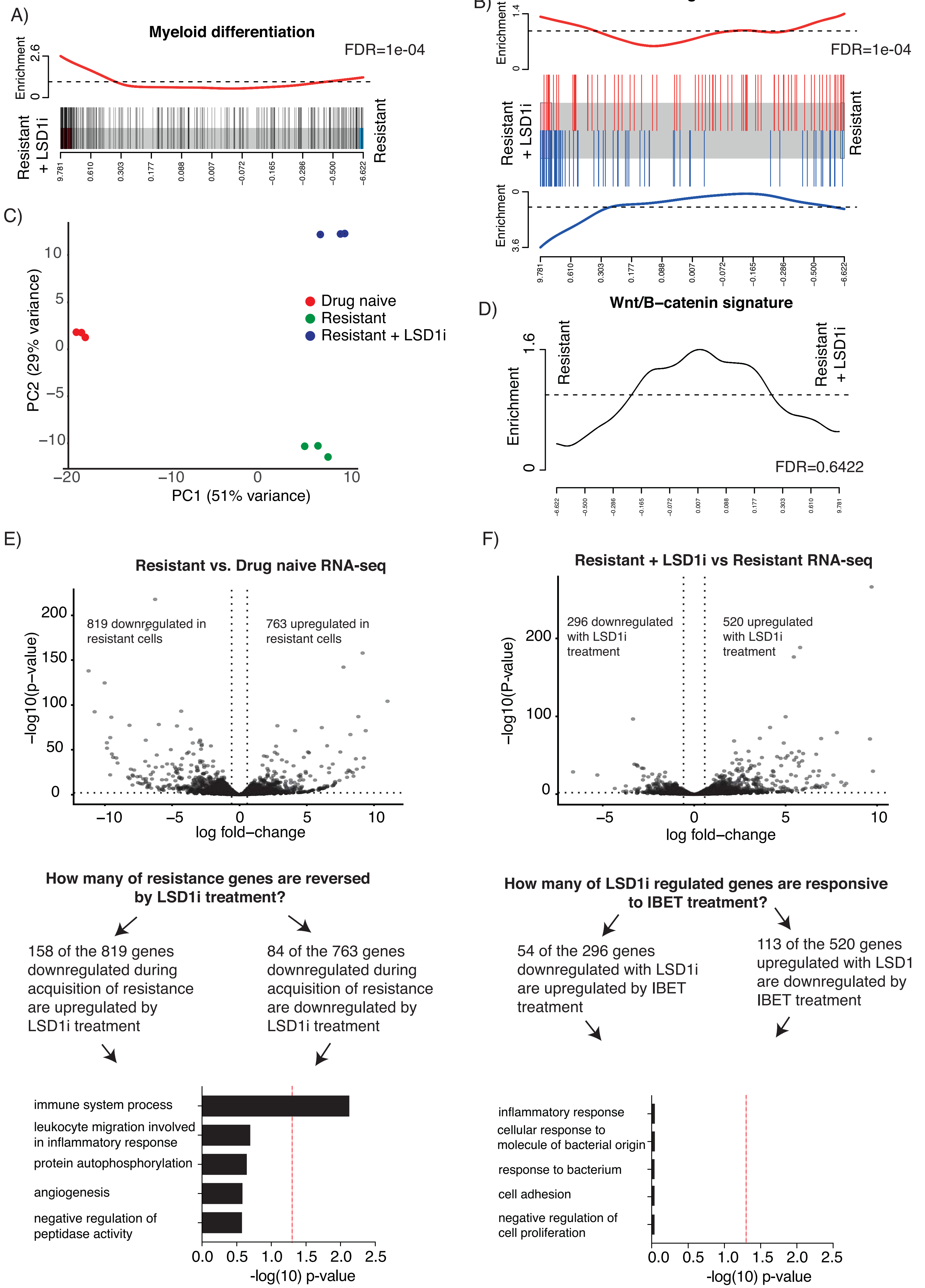

Supplementary Figure 5: **LSD1i treatment causes resensitization without reverting the cells to a drug naïve state**

(A) Gene set enrichment analysis of the genes downregulated during normal myeloid differentiation, comparing resistant cells to resistant cells treated with GSK-LSD1i (500nM) for 6 days. FDR, false discovery rate. **(B)** Gene set enrichment analysis of the LSC gene signature, comparing resistant cells to resistant cells treated with GSK-LSD1i (500nM) for 6 days. Red line indicates enrichment of genes upregulated in the LSC signature. Blue line indicates enrichment of genes downregulated in the LSC signature. FDR = false discovery rate. **(C)** Principal component analysis of bulk RNA-seq data from drug naïve, resistant and resistant cells treated for 6 days with GSK-LSD1i (500nM). **(D)** Gene set enrichment analysis of the Wnt/Beta catenin signature, comparing resistant cells to resistant cells treated with GSK-LSD1i (500nM) for 6 days. FDR, false discovery rate. **(E)** (Top) Volcano plot of RNAseq data comparing drug naïve and resistant cells. (Bottom) Flow chart demonstrating how many of the resistance-associated transcriptional changes are reversed by LSD1i treatment. Resistance associated genes are those that are differentially expression between the resistant and drug naïve populations. Gene Ontology (GO) analysis of these genes identified immune system process as the only enriched biological process. **(F)** (Top) Volcano plot of RNAseq data comparing resistant cells with resistant cells treated for 6 days with GSK-LSD1i (500nM). (Bottom) Flow chart demonstrating how many of the genes that are differentially expressed in the resistant cells after 6 days GSK-LSD1 treatment, become responsive to 1000nM IBET. Gene Ontology (GO) analysis of these genes identified no enriched biological processes associated with these genes

Supplementary Figure 6

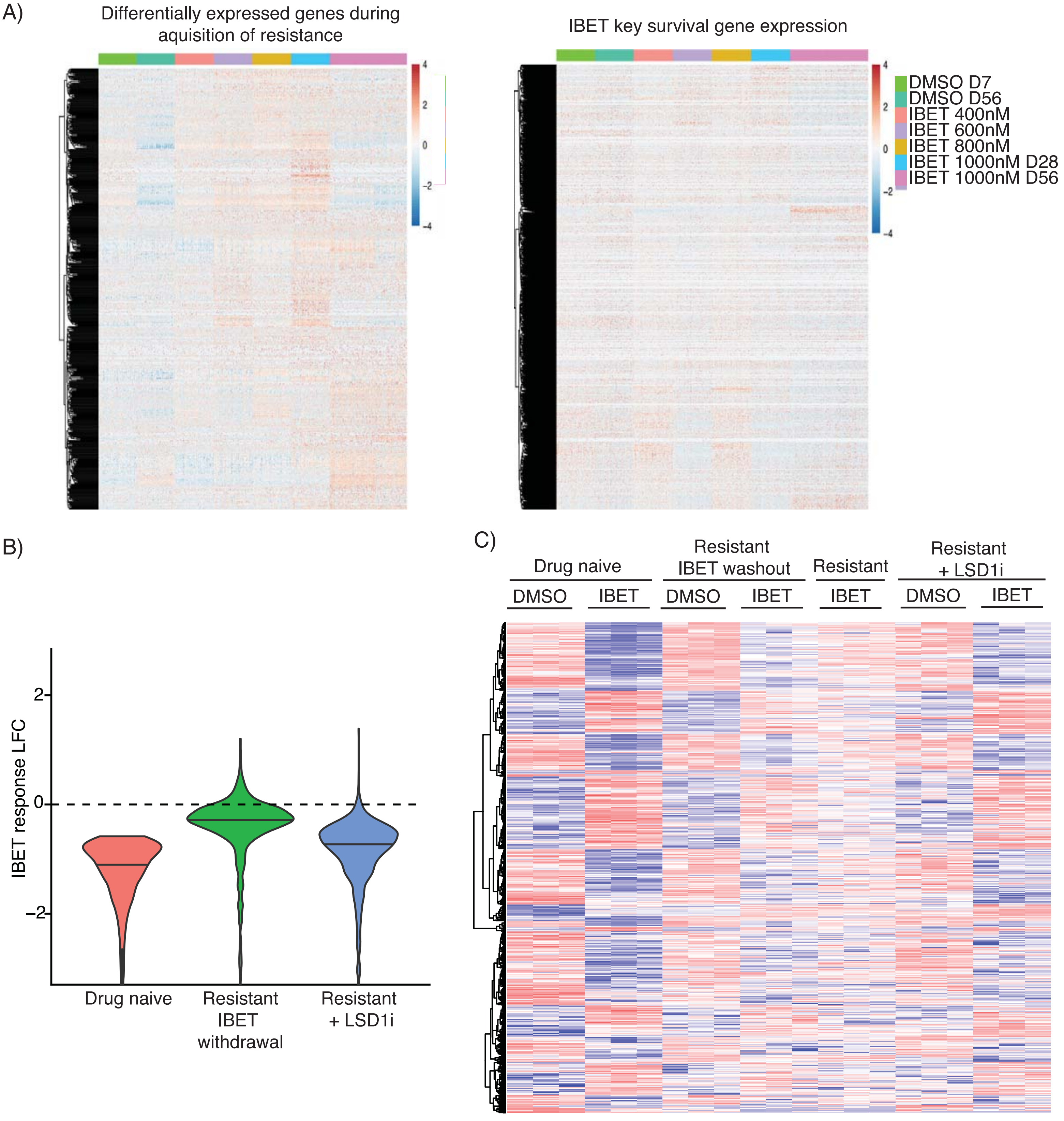

Supplementary Figure 6: LSD1i treatment restores IBET response

(A) Heatmap of the differentially expressed genes between DMSO vs 1000nM IBET samples in single cells across the acquisition of resistance (genes up or downregulated by > 2 FC, p-value<0.01) **(B)** Expression of the IBET key survival genes in single cells across the acquisition of resistance (genes from figure. 3A**,** genes up or downregulated by > 2FC, p-value<0.01) **(B)** Violin plot displaying the response of key survival genes (genes shown in Fig. 3A that are downregulated by > 1.5FC, p-value<0.01 in drug naïve cells after 6hrs of IBET treatment (1000nM)). Shown here is the effects of 6 hours of IBET treatment in drug naïve cells, resistant cells withdrawn from IBET for 6 days and resistant cells pre-treated for 6 days with GSK-LSD1i (500nM). **(C)** RNA-seq heatmap displaying the response of IBET regulated genes (genes up or downregulated by > 1.5FC, p-value<0.01 in drug naïve cells after 6hrs of IBET treatment (1000nM)) in drug naïve cells, resistant cells, resistant cells withdrawn from IBET for 6 days and resistant cells pre-treated for 6 days with GSK-LSD1i (500nM), with or without 6hr IBET (1000nM) treatment.

Supplementary Figure 7

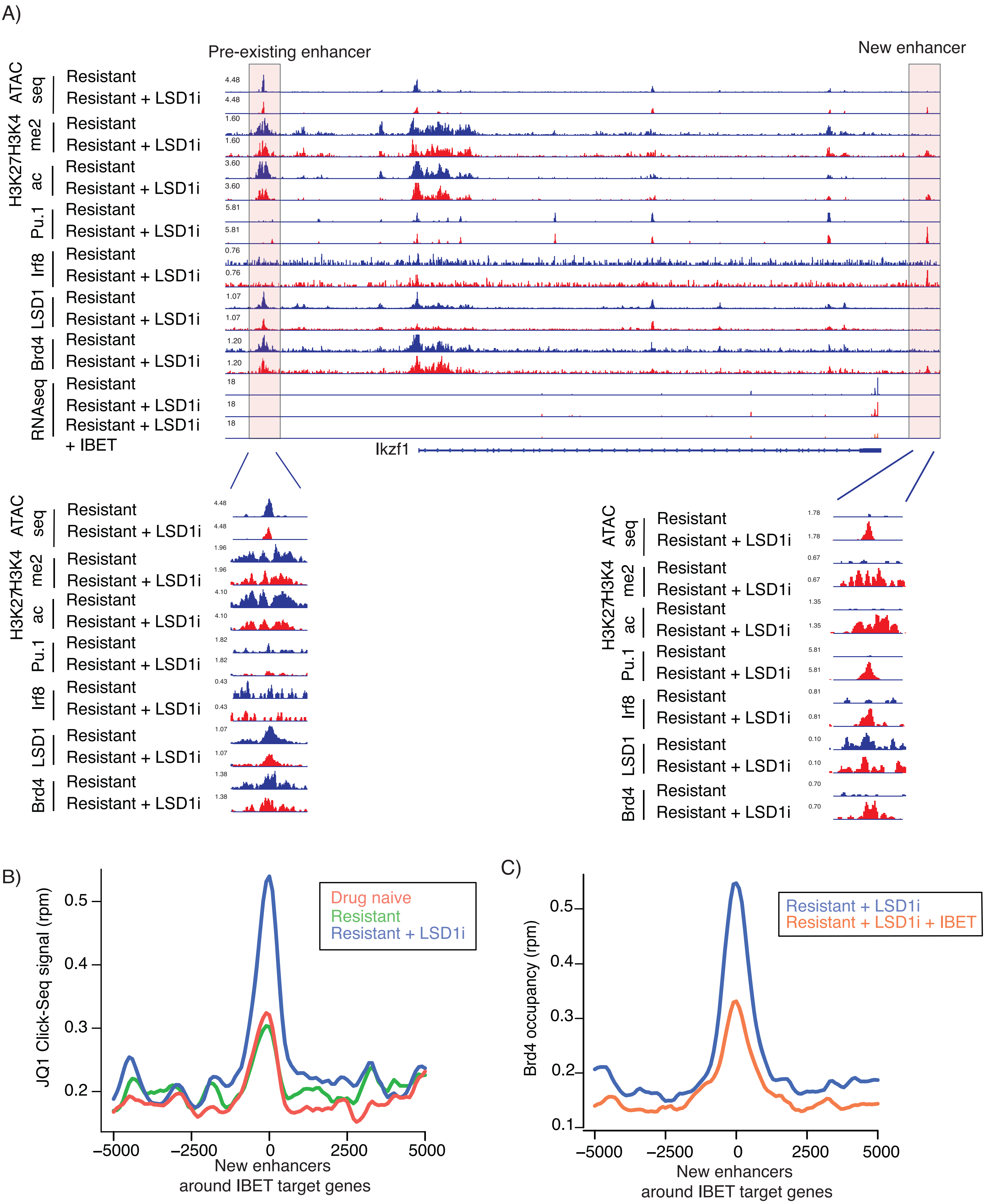

Supplementary Figure 7: Enhancer remodeling occurs around BET inhibitor target genes

(A) Genome browser snapshot from the Integrated Genome Viewer of ATAC-seq and ChIP-seq data around the *Ikzf* locus showing a newly formed active enhancer in resistant cells treated with GSK-LSD1i (500nM) for 6 days. **(B)** JQ1-Click-seq signal at newly activated enhancer elements (as described and shown in Fig. 3B) in drug naïve, resistant and resistant cells treated for 6 days with GSK-LSD1i (500nM). **(C)** Brd4 signal and displacement at newly activated enhancer elements (as described and shown in Fig. 3B) in resistant cells treated for 6 days with GSK-LSD1i (500nM), with or without 6hr IBET treatment.

Supplementary Figure 8

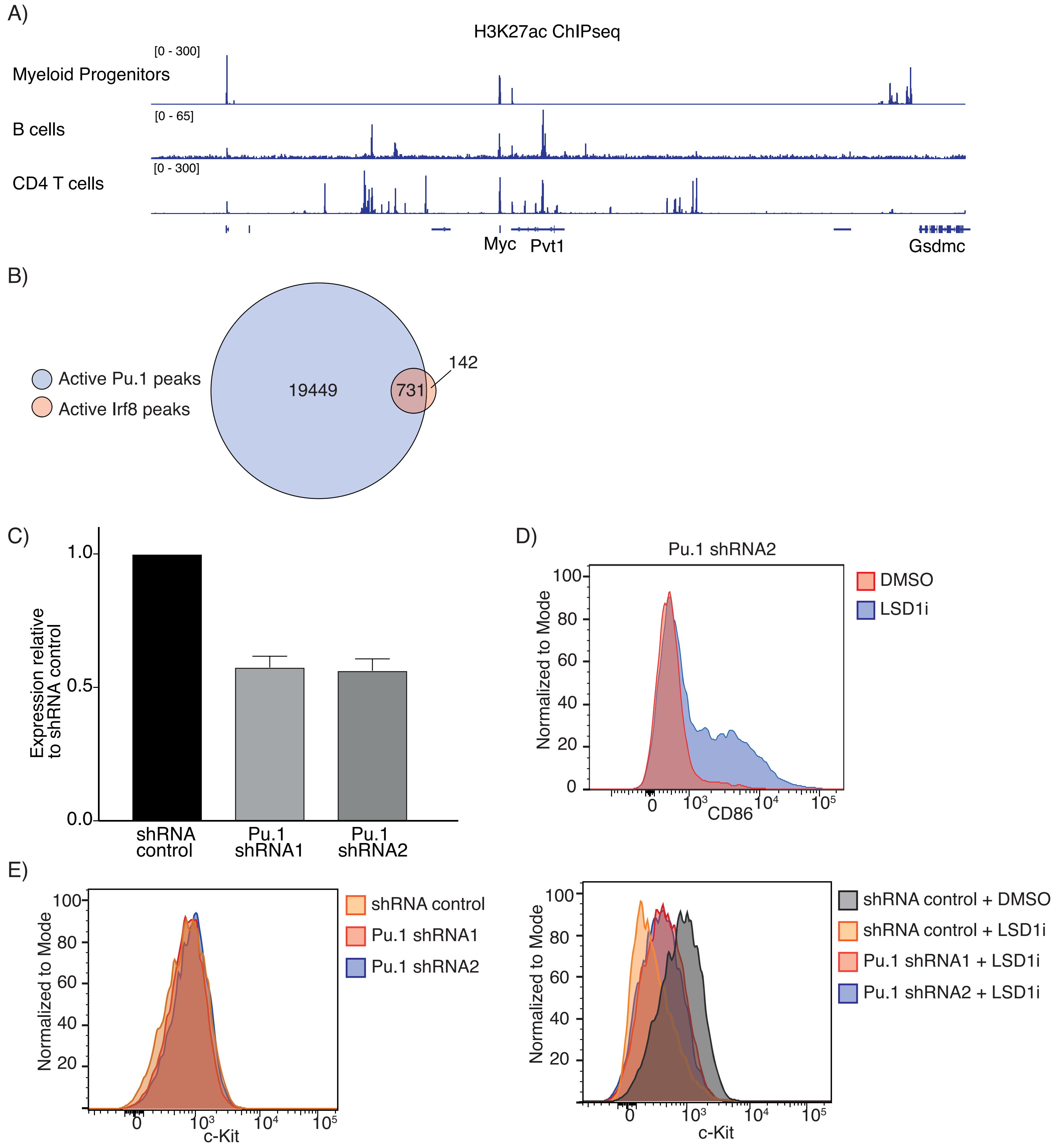

Supplementary Figure 8: Pu.1 is required for differentiation and resensitization

(A) Genome browser snapshot from Integrated Genome Viewer (IGV) of published H3K27ac ChIPseq datasets demonstrating differential enhancer usage between Myeloid Progenitors, B cells and CD4 T-cells at the Myc locus. **(B)** Venn diagram of overlap of active Pu.1 and Irf8 peaks genome-wide. Active enhancers are defined by presence of overlapping H3K27ac peak. **(C)** qPCR analysis of Pu.1 expression levels in shRNA_control and shRNA_Pu.1 resistant cells. qPCR is from 3 biological replicates. **(D)** Flow cytometry of CD86 expression in shRNA_control and shRNA_Pu.1 resistant cells treated for 6 days with DMSO or GSK-LSD1i (500nM). **(E)** Flow cytometry of c-Kit expression in shRNA_control and shRNA_Pu.1 resistant cells treated for 6 days with DMSO (left) or GSK-LSD1i (500nM) (right).

Supplementary Figure 9

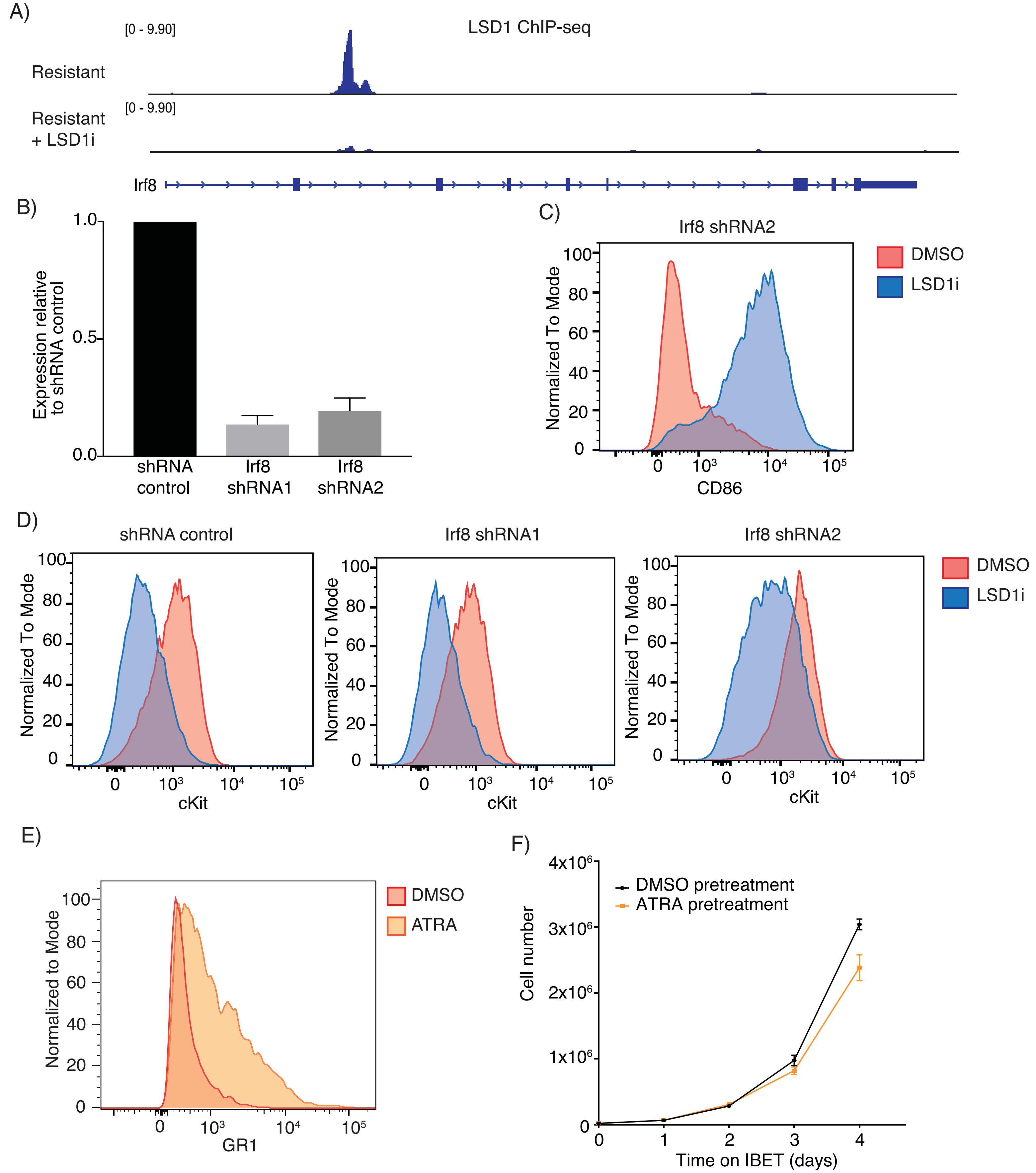

G)

599 LSD1i mediated changes

↓

67 LSD1i mediated changes that are blocked by > 2 fold in Irf8 KD

↓

16 Genes blocked by Irf8 KD that change during the acquisition of resistance (i.e drug naive vs resistant)

S100a9	S100a8	Mgam	Ngp
Hgf	Cd300lf	Bmx	Thbs1
Clec5a	Gm38299	F13a1	Milr1
Flt1	Depp1	Depdc7	Rgs3

Supplementary Figure 9: Irf8 is a direct LSD1 target and is dispensable for differentiation

(A) Genome browser snapshot from the Integrated Genome Viewer of LSD1 occupancy at the Irf8 locus in resistant cells and resistant cells treated for 3hrs with GSK-LSD1i (500nM). **(B)** qPCR analysis of Irf8 expression levels in shRNA control and Irf8 shRNA resistant cells. qPCR is from 3 biological replicates. **(C)** Flow cytometry of CD86 expression in shRNA_control and shRNA_Irf8 resistant cells treated for 6 days with DMSO or GSK-LSD1i (500nM). **(D)** Flow cytometry of c-Kit expression in shRNA_control and shRNA_Irf8 resistant cells treated for 6 days with DMSO or GSK-LSD1i (500nM). **(E)** Flow cytometry of GR1 expression in resistant cells treated for 6 days with DMSO or ATRA (1000nM). **(F)** Proliferation assay of resistant cells pre-treated for 6 days with DMSO or ATRA (1000nM) followed by treatment with IBET (1000nM). Error bars represent S.E.M of 3 cell culture replicates. Representative of 3 biological replicates. **(G)** (Top) Number of LSD1i induced transcriptional changes in shRNA_control cells treated for 6 days with GSK-LSD1i. (Middle) Number of these LSD1i induced gene expression changes (from top) that are blocked (by at least 2 fold) in Irf8 shRNA resistant cells. (Bottom) Number of the Irf8 KD blocked changes (from middle) that are differentially expressed during the acquisition of resistance. (i.e. genes that are differentially expressed between drug naïve and resistant cells).

Supplementary Figure 10

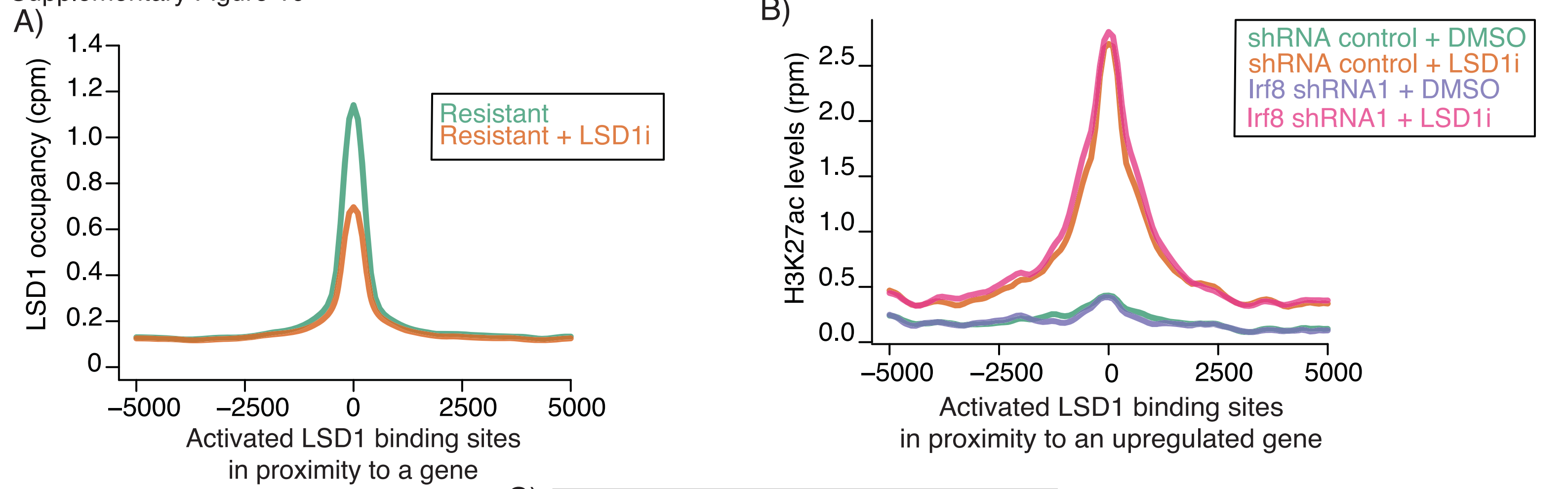

C)

**Proportion of genes with an activated LSD1 binding site**	
Up-regulated genes	323/520
Down-regulated genes	19/296
Not differentially expressed	1055/22181

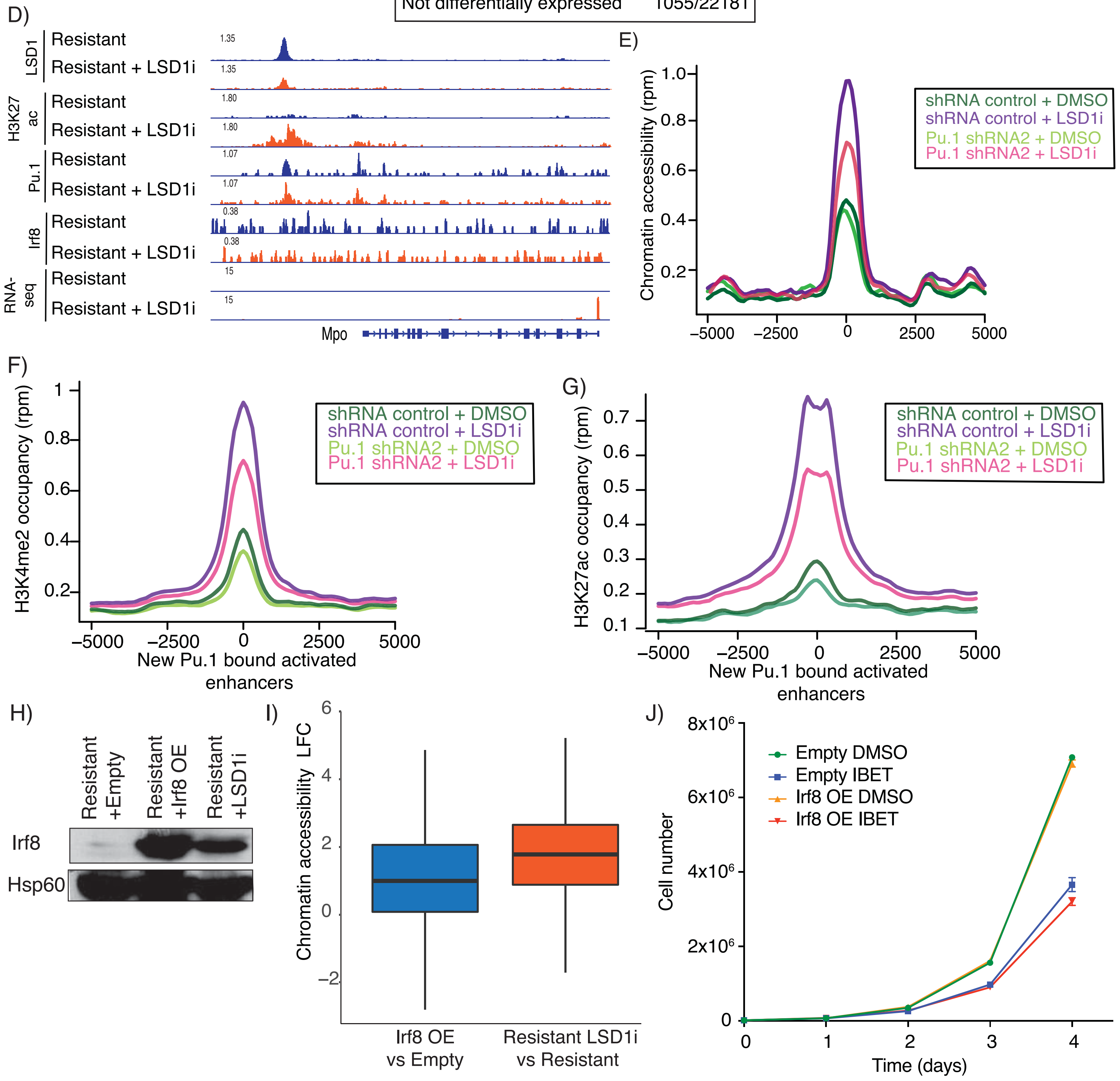

Supplementary Figure 10: **LSD1 inhibitor direct de-represses differentiation associated genes.**

(A) Average profile of LSD1 occupancy at activated LSD1 peaks (>2 fold increase in H3K27ac after 6 days LSD1i treatment) located within 10kb of any gene. Displayed are the levels of LSD1 at these loci in resistant cells and resistant cells treated for 3hrs with LSD1i. **(B)** Average profile of H3K27ac levels at activated LSD1 peaks around LSD1i up-regulated genes in shRNA control and shRNA_Irf8 resistant cells treated with DMSO or LSD1i for 6 days. **(C)** Number of activated LSD1 binding sites in proximity to LSD1i upregulated, downregulated or non-regulated genes. **(D)** Genome browser snapshot from the Integrated Genome Viewer at the *Mpo* locus in resistant cells and resistant cells treated for 3hrs with GSK-LSD1. This is a representative example of a gene directly de-repressed as a result of LSD1i treatment. **(E)** Average profile of chromatin accessibility in resistant cells expressing shRNA_control and Pu.1 shRNA2 at sites of increased Pu.1 binding (>4 fold increase) after 6 days treatment with either DMSO or GSK-LSD1i (500nM). **(F)** Average profile of H3K4me2 occupancy in resistant cells expressing shRNA control and Pu.1 shRNA2 at sites of increased Pu.1 binding (>4 fold increase) after 6 days treatment with either DMSO or GSK-LSD1i (500nM). **(G)** Average profile of H3K27ac occupancy in resistant cells expressing shRNA control and Pu.1 shRNA2 at sites of increased Pu.1 binding (>4 fold increase) after 6 days treatment with either DMSO or GSK-LSD1i (500nM). **(H)** Western blot of Irf8 levels in resistant cells containing empty vector or Irf8 overexpression cDNA constructs. Irf8 levels in resistant cells pretreated with GSK-LSD1i (500nM) for 6 days is shown for reference. **(I)** Box plot of changes in chromatin accessibility in resistant cells overexpressing Irf8 treated with DMSO for 6 days or resistant cells overexpressing the empty vector control treated for 6 days with GSK-LSD1i (500nM). Chromatin accessibility was measured at newly activated Pu.1 enhancers (>4 fold increase H3K27ac and Pu.1 binding). **(J)** Proliferation assay of empty vector control or Irf8 overexpression in resistant cells grown in DMSO or IBET (1000nM). Error bars represent S.E.M of 3 cell culture replicates. Representative of 3 biological replicates.

Supplementary Note 1:

Mathematical Modelling

We used a discrete-time dynamical system of tumour growth to find the proliferation rates of sensitive and resistant subpopulations, similar to the approach of Misale et al. [2]. This model was based on the population mixture observed from single-cell RNA sequencing (Fig 1A and B) and we used estimates of malignant cell counts based on bone marrow blast count percentages collected from patients during disease progression (Fig 1A and B) scaled to the assumption of a lethal size of one trillion cells previously reported for leukaemia [1].

Using this framework, we estimate that if a resistant subpopulation originated during BET inhibitor treatment, its resulting proliferation rate b_r^T would be ~0.60 in both patients, a 10% increase from baseline. According to Bozic et. al. [3] the average selective advantage conferred by driver mutations is 0.004 ± 0.0004, which means such an elevated proliferation rate would require accumulation of at least 23 driver alterations in a short period of time. Using the same framework we estimated that τ, the time required to achieve an overall 10% selective advantage, would result in resistance taking ~14 times longer to emerge than was observed. With the estimated values it is unlikely that a drug resistance mutation acquired during IBET treatment swept to the observed growth kinetics in an interval of ~100-200 days.

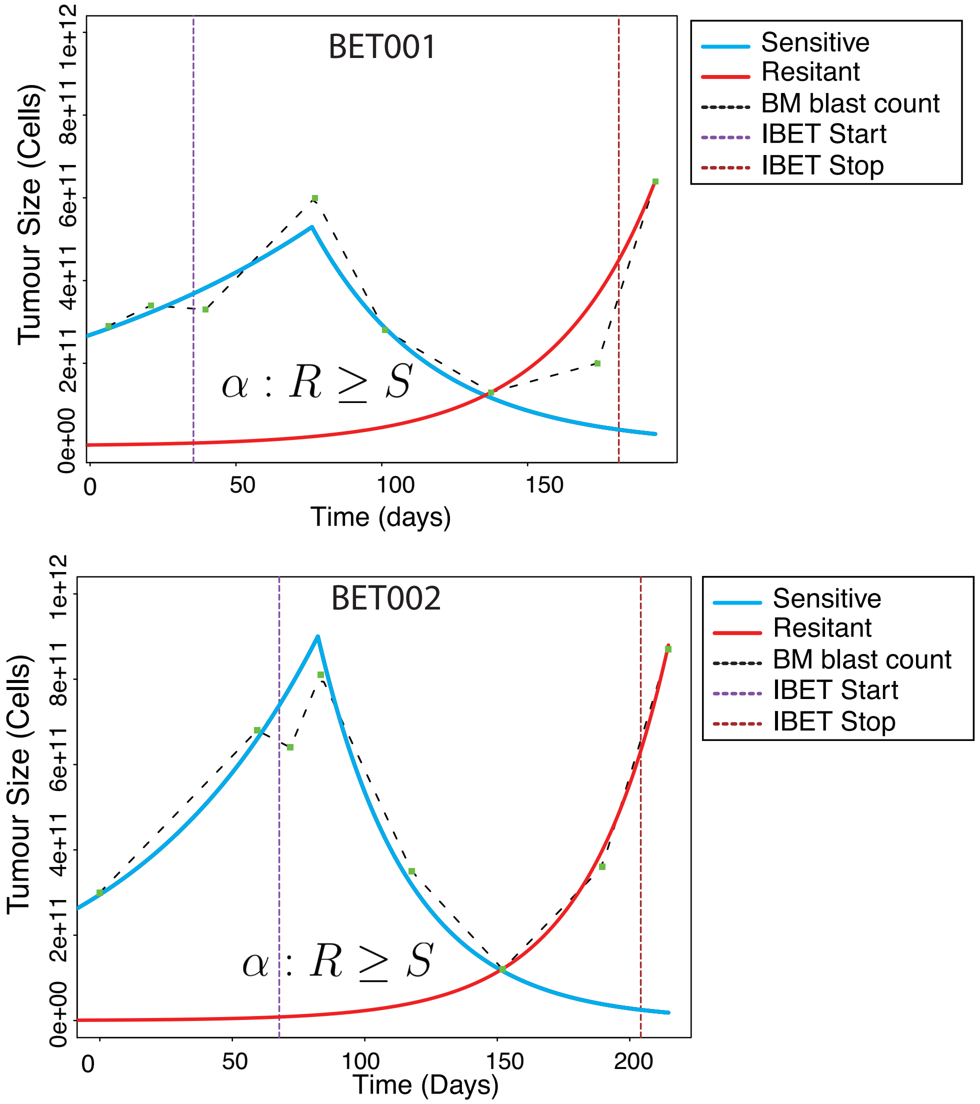

Supplementary Figure 11: Fitting Output*.* *Estimated growth trajectories of the sensitive (blue) and resistant (red) subpopulations between the first and last sampled time points, for each patient. The total number of tumour cells at each time point as estimated from blast counts are given as green squares. Both patients showed similar proliferation rates in the sensitive sub-population (blue) and in the drug-resistant resistant (red).*

**Patient**	$R_{t=b}$	b_s	b_r	RMSE	b_r^T	τ (Days)
BET001	*1.4*	*0.505*	*0.515*	*0.22*	*0.595*	*2201*
BET002	*0.93*	*0.507*	*0.517*	*0.32*	*0.605*	*2247*

Supplementary Table 1: Best fits

$R_{t=b}$: Percentage of pre-existing resistant cells at first sequence snapshot, RMSE: root mean square error, b_r^T: Proliferation rate of resistance subpopulation if originated under treatment, τ: Expected time to have an increase of 10% of growth rate assuming division time of 1 day

As shown in **Supplementary Figure 11** and **Supplementary Table 1** (Best Fits), the best fits for both patients show higher proliferation rates for the resistant subpopulations over the drug sensitive subpopulations and the presence of resistant cells prior to BET inhibitor therapy. Our modelling estimates the proportion of resistant cells capable of non-genetic adaptation prior to drug exposure at approximately 1-2%. This is concordant with t-SNE clustering of single-cell expression profiles (Fig. 1 A and B) and published *in vitro* results of Pisco et al [4], who found subpopulations within leukemia cell lines harbouring epigenetic drug resistance mechanisms at frequencies similar to what we estimate existed in our patients prior to BET inhibitor treatment.

A summary of the fitting procedure is shown in Supplementary Figure 12.

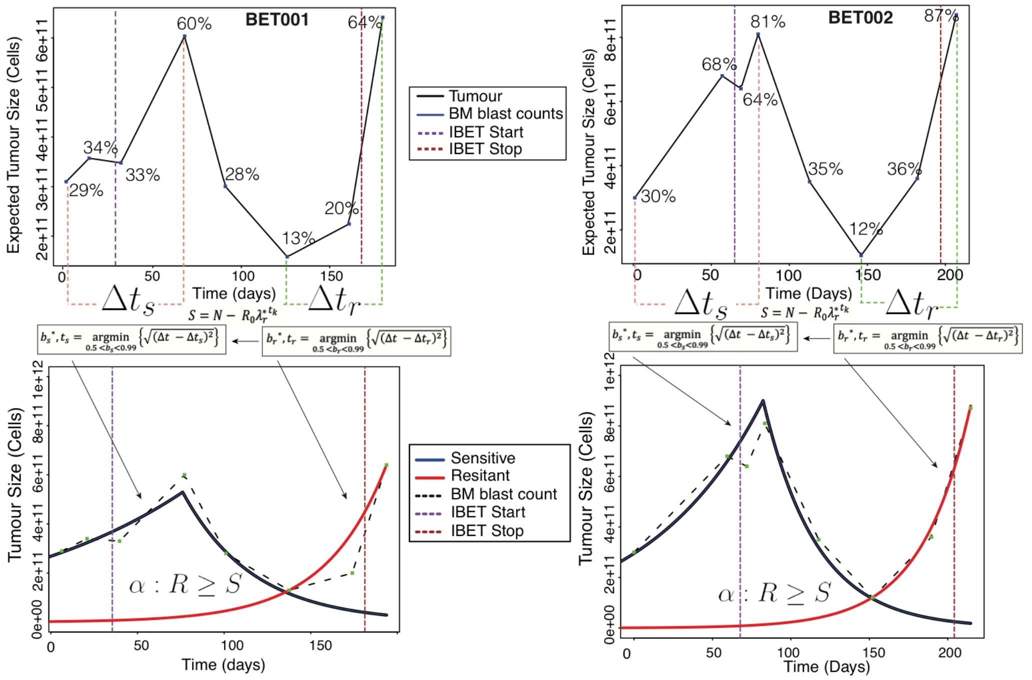

***Supplementary Figure 12**: a) The proliferation rate of drug resistant cells (R) is obtained using the interval Δt_r from minimum blast count (i.e., maximum remission) to the final post-IBET blast count (red). Similarly, we obtain the proliferation rate of drug sensitive cells (S) by fitting to blast count data in the interval Δt_s from the first pre-BET inhibitor sample to start of objective response to the drug (blue). b) b_r^* is used to estimate the proportion of S and R at blast count points, with S inferred through b_s^* using Δt_s. c) Based parameters b_r^* and b_s^* we reconstruct the growth curves, applying a constraint to S at minimum blast count such that α: $R \geq S$.*

The number of tumour cells N at time t is given by,

$$N_t = S_t + R_t \qquad \text{Eq. 1}$$

Where sensitive S and resistant R subpopulations grow exponentially with their corresponding proliferation rates b and death rate d subject to $b > d$ and $b + d = 1$.

$$S_t = S_0(1 + b_s - d_s)^{t-1} \qquad \text{Eq. 2}$$

$$R_t = R_0(1 + b_r - d_r)^{t-1} \qquad \text{Eq. 3}$$

During exponential growth, the time when the population attains a size X with parameters b and d is given by,

$$t = \frac{\log(X)}{\log(X_0(1+b-d))} \qquad \text{Eq. 4}$$

Assuming that type R cells grow in the presence of BET inhibitor and the minima of the estimated number of tumour cells in the curves in Fig. 1 (A and B) correspond to $R \geq S$ outgrowth, we use the previous equation to obtain proliferation rates of R and S with the following fitting procedure.

1. Obtain Δt_R from the observed time difference between the last and the minimum blast count.

2. Using Eq. 3, fit the drug resistance curve (b_r^* and t_r) by minimising the error of the observed time interval.

$$b_r{}^*, t_r = \underset{0.5 < b_r < 0.99}{\operatorname{argmin}} \left\{ \sqrt{\left(\frac{\log{(R_f)}}{\log{(\lambda_r)}} - \frac{\log(R_i)}{\log(\lambda_r)} - \Delta t_r \right)^2} \right\},$$

3. Scale subject to b_r^* and t_r, estimate the proportion of sensitive and resistant cells at blast counts measurements time points with the following general form,

$$R = R_0(1 + b_r^* - d_r)^{t_r - t_i},$$

$$S = N - R,$$

4. Obtain Δt_S from the observed blast count time difference between the initial and the maximum before population crash.

5. Similar to step 2, fit the drug sensitive curve ($b_s{}^*$ and t_S) by minimising the error of the observed time window using the scaled measurements from step 3.

6. Adjust time indexes and use b_r^* and b_s^* to simulate from $t = 1 \ldots N$ penalising the sensitive sub-population with α during the decay, compute the root mean square error for all observations.

6.a Apply a penalty α to S from the point at which blast counts decrease until end of BET inhibitor treatment, such that $R \geq S$ at the minima,

$$S_t = S_{t-1}(1 + b_s - d_s) - S_{t-1}(1 - b_s - d_s)\alpha,$$

$$\{b_s{}^*, b_r{}^*, \alpha^*\} = \underset{0.01 < \alpha < 0.7}{\operatorname{argmin}} \left\{ \sqrt{\frac{1}{n} \sum_{i=1}^{n} \left(\frac{N_i}{M} - \frac{\widehat{N_\iota}}{M} \right)^2} \right\},$$

Definitions

S_t	Size of sensitive population at time t
R_t	Size of resistant population at time t
b_s	Proliferation rate of sensitive
d_s	Death rate of sensitive
b_r	Proliferation rate of resistant
d_r	Death rate of resistant
N_t	Tumour size at time t
R_0	Proportion of resistant cells at initial blast count
X	Blast count
Δt	Difference in time from blast counts
$t_r - t_i$	Time adjustment relative to the total simulated time of R
α	Penalty for drug sensitive decay
λ	General form of rate of growth $N_0(1 + b - d)$

Supplementary References

[1] Aroesty, J., et al. "Tumor growth and chemotherapy: mathematical methods, computer simulations, and experimental foundations." *Mathematical Biosciences* 17.3-4 (1973): 243-300.

[2] Misale, Sandra, et al. "Vertical suppression of the EGFR pathway prevents onset of resistance in colorectal cancers." *Nature communications* 6 (2015): 8305.

[3] Bozic, Ivana, et al. "Accumulation of driver and passenger mutations during tumor progression." *Proceedings of the National Academy of Sciences* 107.43 (2010): 18545-18550

[4] Pisco, Angela Oliveira, et al. "Non-Darwinian dynamics in therapy-induced cancer drug resistance." *Nature communications* 4 (2013): 2467.

Figure 5E

Pu.1 WB

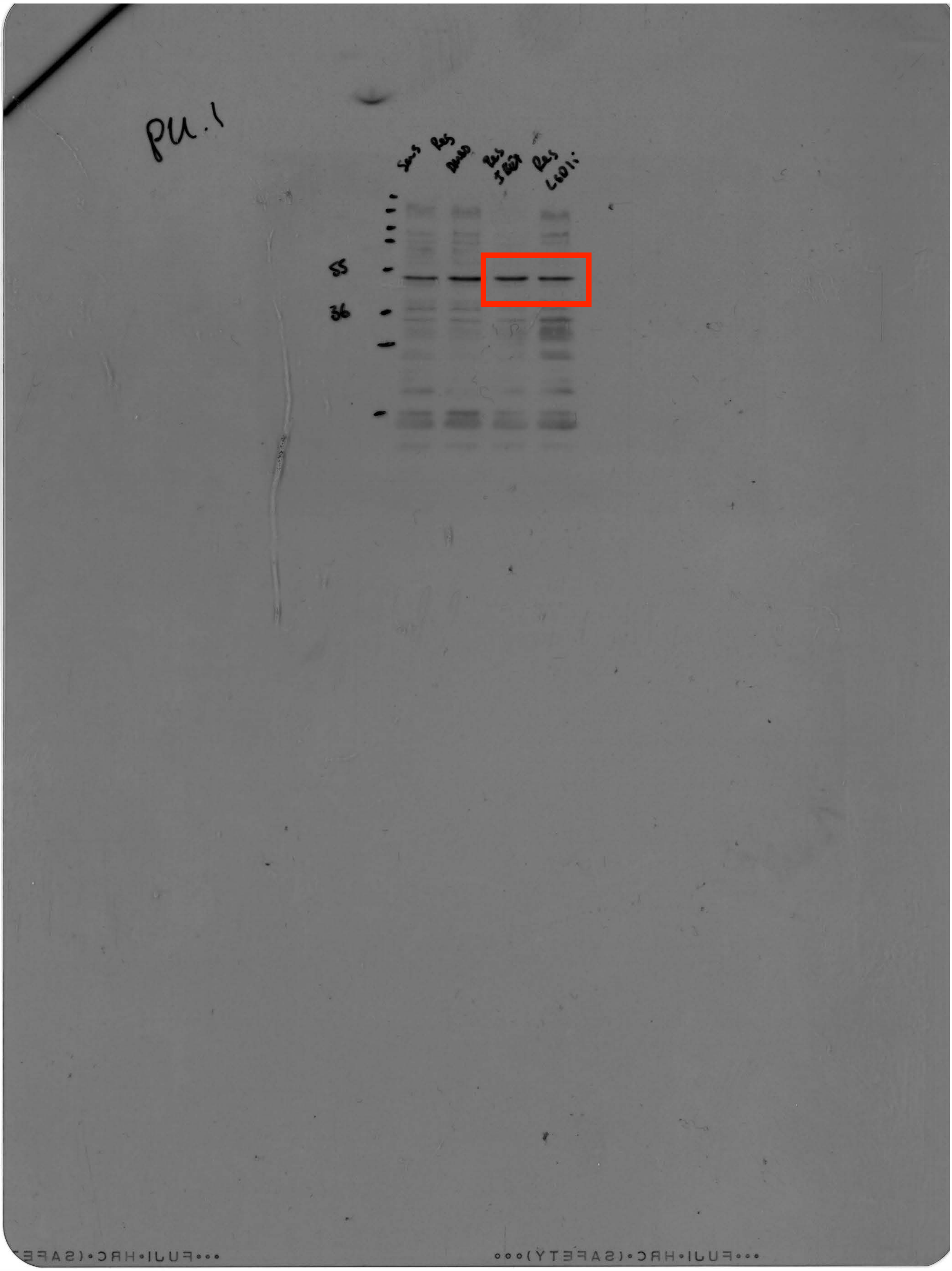

Figure 5E

HSP60 WB

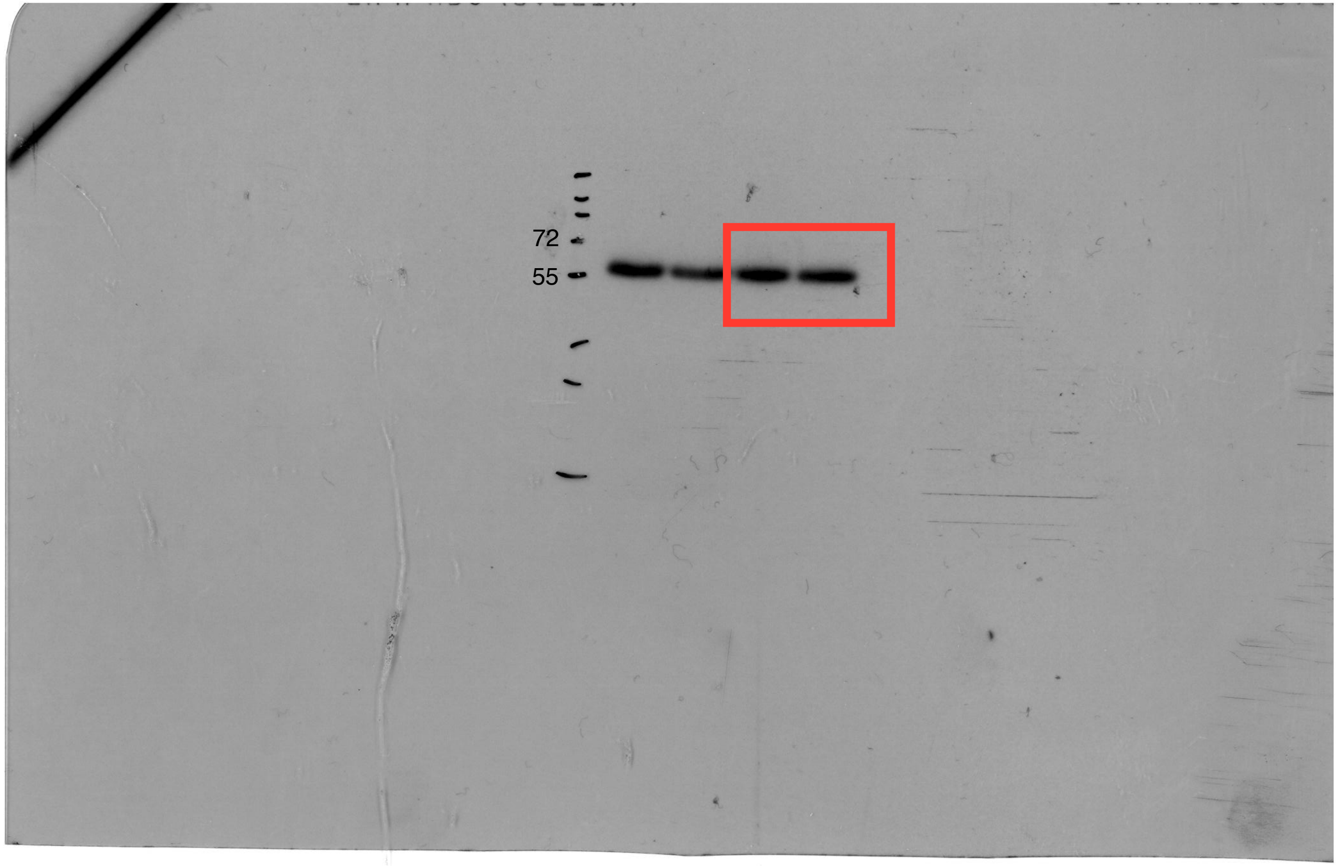

Figure 5F

Irf8 WB
High exposure

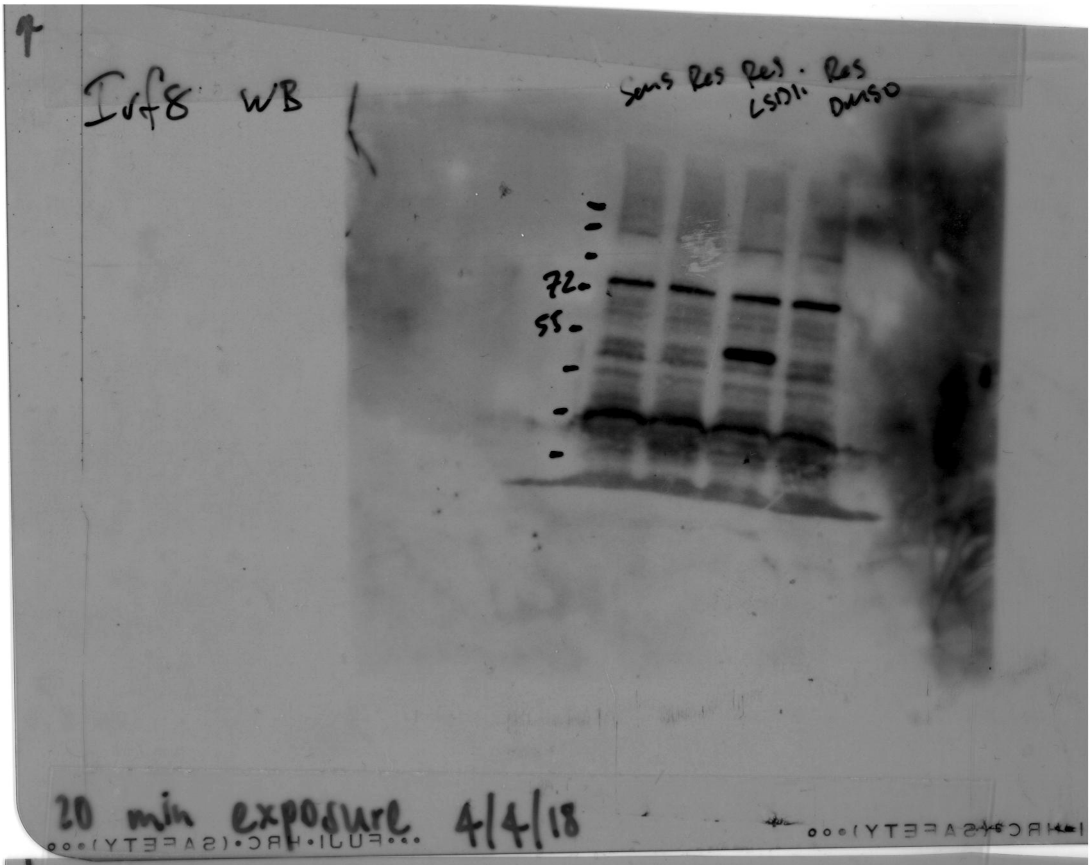

Figure 5F
Irf8 WB
Low exposure

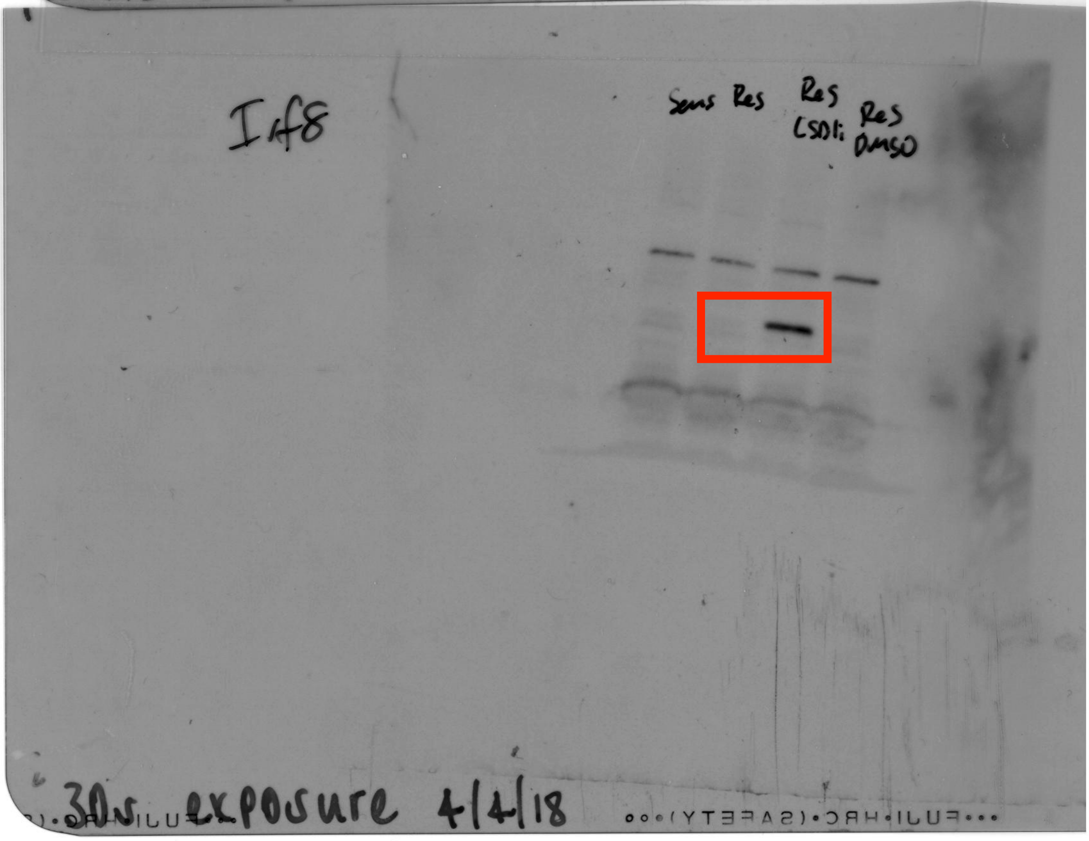

Figure 5F
HSP60
WB

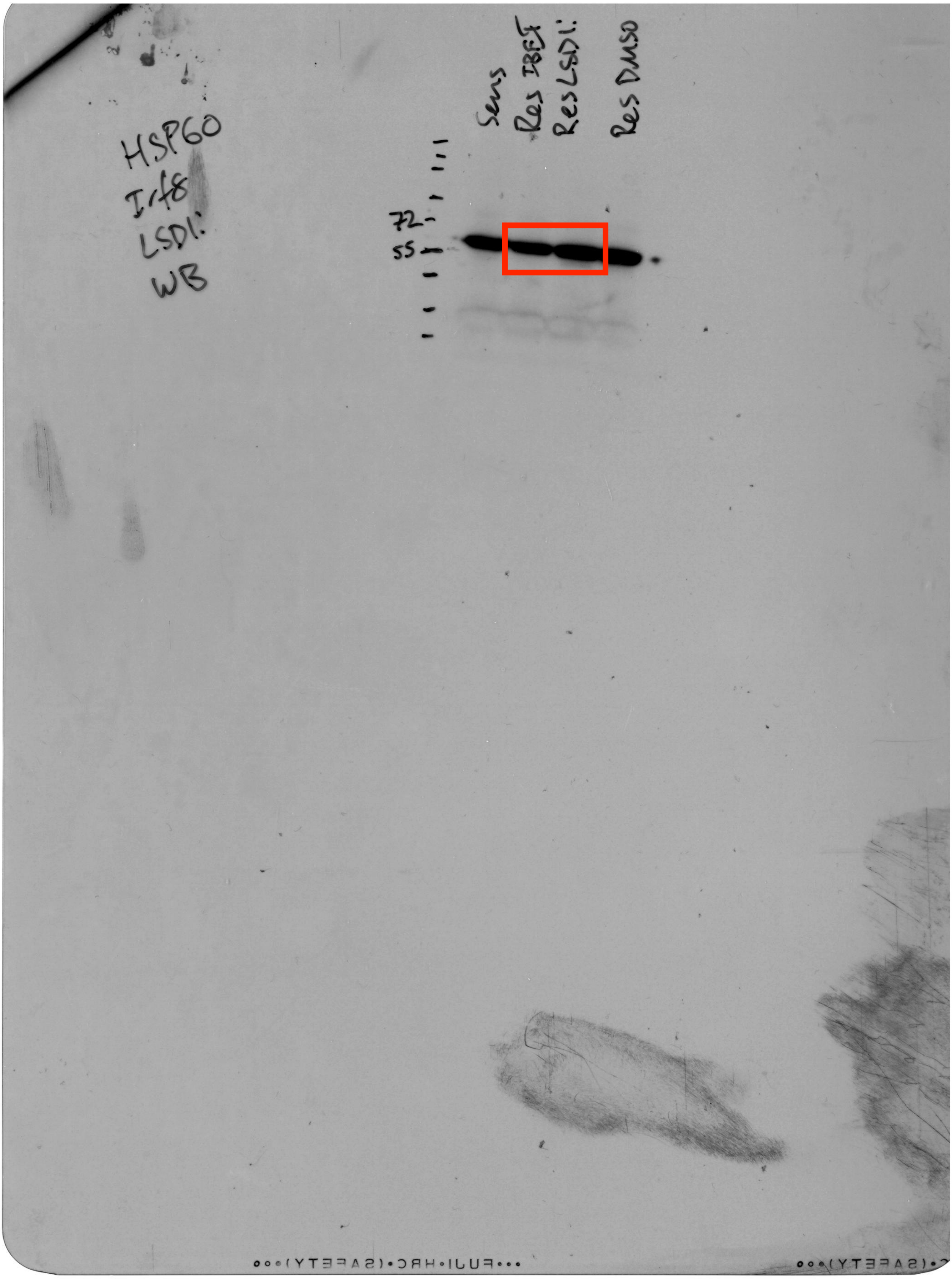

Supplementary Figure 4A
HSP60 and LSD1 WB

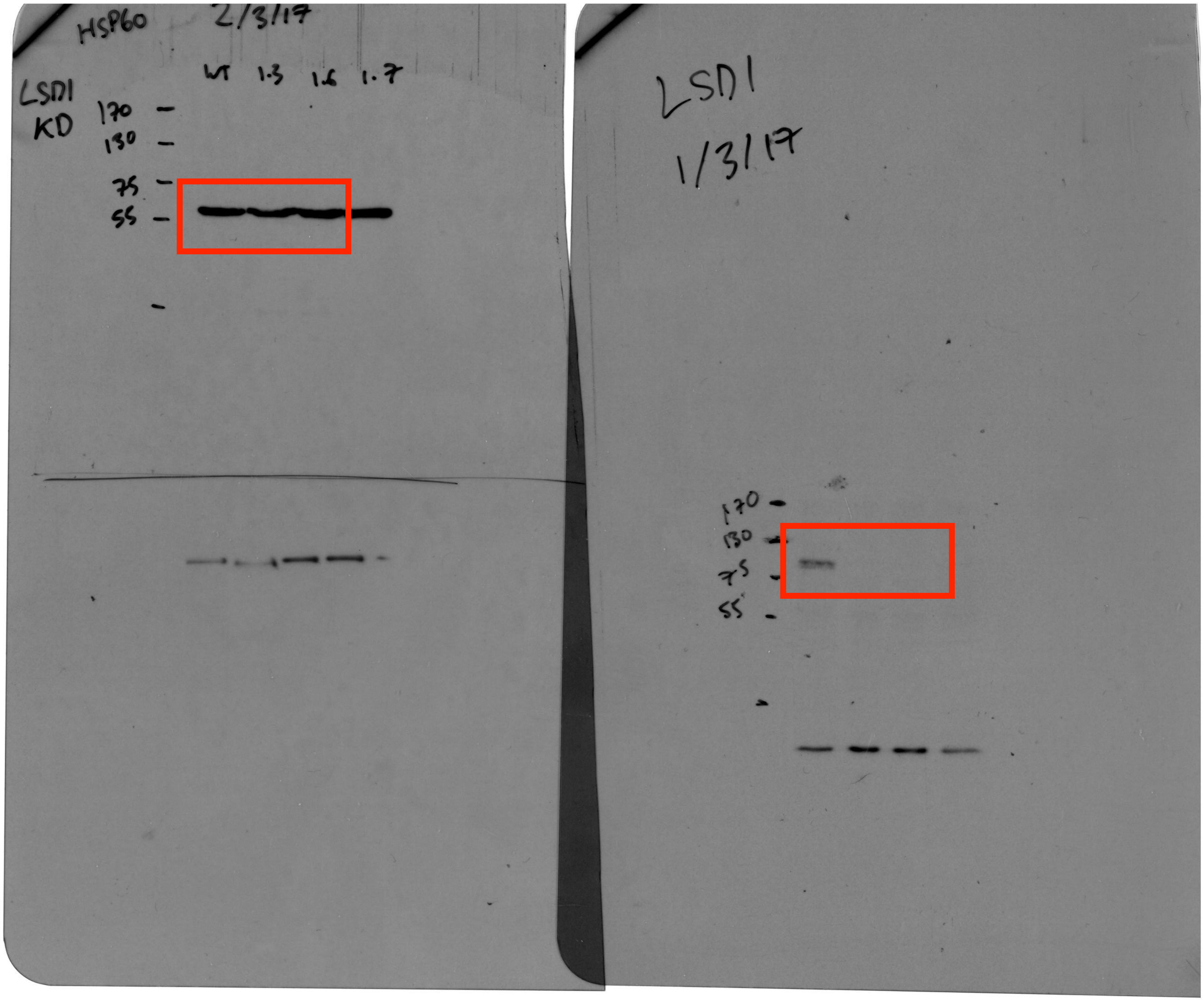

Supplementary Figure 10H

Irf8 WB

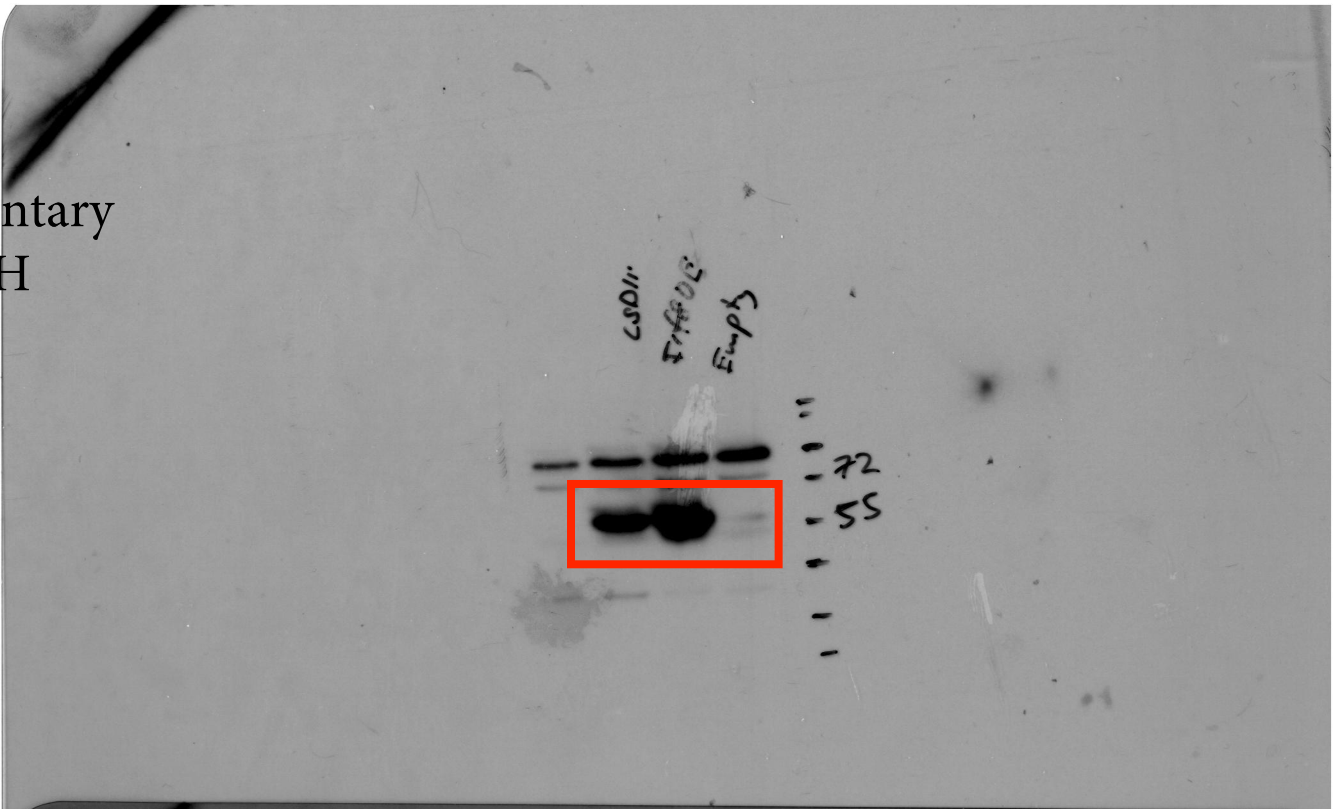

Supplementary Figure 10H

Hsp60 WB

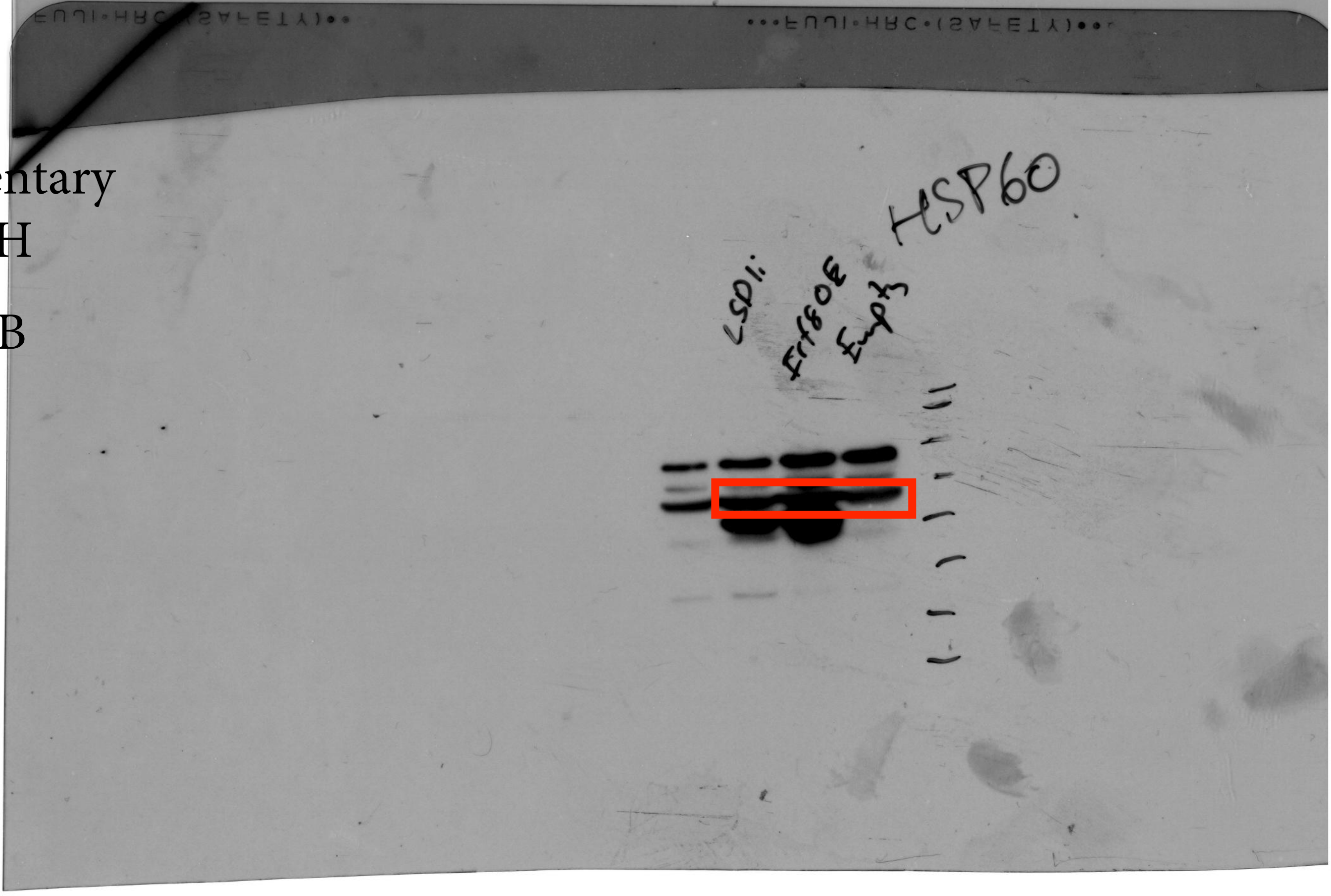

Supplement: Supplementary file 1 — Supplementary Information [file 41467_2019_10652_MOESM1_ESM.pdf]
