# Supplementary material for: Targeting enhancer switching overcomes non-genetic drug resistance in acute myeloid leukaemia
Source: Nat Commun. 2019 Jun 20;10:2723. doi: 10.1038/s41467-019-10652-9 (PMC6586637; doi:10.1038/s41467-019-10652-9)
Supplement: Supplementary file 3 — Description of Additional Supplementary Files [file 41467_2019_10652_MOESM3_ESM.pdf]

Description of Additional Supplementary Files

File name: Supplementary Data 1

Description: Table of sgRNA names and their corresponding fold enrichment in the GR1+ and GR1- populations.

Table is ranked by enrichment in the GR1+ population: When assessed by relative enrichment (GR1+/GR1-) the following genes have the most (≥ 3) independent sgRNA's enriched 5-fold: Setdb1; Chd4; Hbo1; Hdac3 and Kdm1a. In contrast, when assessed by relative enrichment of GR1-/GR1+ the following genes have the most (≥ 3) independent sgRNA's enriched 5-fold: Mll4; Kdm6a and Crebbp.

File name: Supplementary Data 2

Description: List of key survival genes from Fig. 4A

Table demonstrates the significantly downregulated genes in drug naïve cells after 6hrs IBET treatment (1000nM). Ranked by most downregulated in response to IBET. Positive log fold change = genes upregulated by IBET treatment, negative log fold change = genes downregulated by IBET treatment.

File name: Supplementary Data 3

Description: List of primer and shRNA sequences

Table of forward and reverse primer sequences for qPCR and sequencing and the shRNA sequences along with their respective target.
