# Supplementary material for: Targeting enhancer switching overcomes non-genetic drug resistance in acute myeloid leukaemia
Source: Nat Commun. 2019 Jun 20;10:2723. doi: 10.1038/s41467-019-10652-9 (PMC6586637; doi:10.1038/s41467-019-10652-9)
Supplement: Supplementary file 7 — Reporting Summary [file 41467_2019_10652_MOESM7_ESM.pdf]

nature research

Corresponding author(s): Mark Dawson

Last updated by author(s): Apr 24, 2019

Reporting Summary

Nature Research wishes to improve the reproducibility of the work that we publish. This form provides structure for consistency and transparency in reporting. For further information on Nature Research policies, see Authors & Referees and the Editorial Policy Checklist.

Statistics

For all statistical analyses, confirm that the following items are present in the figure legend, table legend, main text, or Methods section.

n/a	Confirmed	
☐	☒	The exact sample size (*n*) for each experimental group/condition, given as a discrete number and unit of measurement
☐	☒	A statement on whether measurements were taken from distinct samples or whether the same sample was measured repeatedly
☐	☒	The statistical test(s) used AND whether they are one- or two-sided<br>*Only common tests should be described solely by name; describe more complex techniques in the Methods section.*
☒	☐	A description of all covariates tested
☐	☒	A description of any assumptions or corrections, such as tests of normality and adjustment for multiple comparisons
☐	☒	A full description of the statistical parameters including central tendency (e.g. means) or other basic estimates (e.g. regression coefficient) AND variation (e.g. standard deviation) or associated estimates of uncertainty (e.g. confidence intervals)
☐	☒	For null hypothesis testing, the test statistic (e.g. *F*, *t*, *r*) with confidence intervals, effect sizes, degrees of freedom and *P* value noted<br>*Give P values as exact values whenever suitable.*
☒	☐	For Bayesian analysis, information on the choice of priors and Markov chain Monte Carlo settings
☒	☐	For hierarchical and complex designs, identification of the appropriate level for tests and full reporting of outcomes
☒	☐	Estimates of effect sizes (e.g. Cohen's *d*, Pearson's *r*), indicating how they were calculated

Our web collection on statistics for biologists contains articles on many of the points above.

Software and code

Policy information about availability of computer code

Data collection	All software used for data collection is listed in the Methods. No custom programs were developed specifically for this manuscript.
Data analysis	All software used for data analysis is listed in the Methods. No custom programs were developed specifically for this manuscript.

For manuscripts utilizing custom algorithms or software that are central to the research but not yet described in published literature, software must be made available to editors/reviewers. We strongly encourage code deposition in a community repository (e.g. GitHub). See the Nature Research guidelines for submitting code & software for further information.

Data

Policy information about availability of data

All manuscripts must include a data availability statement. This statement should provide the following information, where applicable:

- Accession codes, unique identifiers, or web links for publicly available datasets
- A list of figures that have associated raw data
- A description of any restrictions on data availability

Data availability statement

The sequencing data that support the findings of this study has been deposited into the sequence read archive, which is hosted by the National Centre for Biotechnology Information. The GEO accession number is GSE110901. The source data underlying Figure 2E, 3G, 5E, 5F, Supplementary Figure 4A, 4D and 10H are provided as a source data file. The remaining source data is available from the authors upon request.

Field-specific reporting

Please select the one below that is the best fit for your research. If you are not sure, read the appropriate sections before making your selection.

☒ Life sciences ☐ Behavioural & social sciences ☐ Ecological, evolutionary & environmental sciences

For a reference copy of the document with all sections, see nature.com/documents/nr-reporting-summary-flat.pdf

Life sciences study design

All studies must disclose on these points even when the disclosure is negative.

Sample size	The number of mice assigned to each treatment arm was selected to provide sufficient statistical power to discern significant differences. The sample size was selected prior with no prior knowledge of the expected outcome.
Data exclusions	Mice were excluded from the Kaper-Meier curves if they had less than 20% detectable leukemic cells in the bone marrow or spleen at the time of death.<br><br>The only other data points excluded were those that were clear outliers in technical replicates of proliferation assays or qRT-PCR.
Replication	All in vitro functional and phenotypic experiments were performed in at least biological triplicate to ensure reproducibility. All experiments were able to be reliably reproduced.
Randomization	Allocation of mice to different groups was random.
Blinding	Experimenters were not blinded to the experiment. However, treatment of mice was performed by the core facility who have no direct involvement in the project.

Reporting for specific materials, systems and methods

We require information from authors about some types of materials, experimental systems and methods used in many studies. Here, indicate whether each material, system or method listed is relevant to your study. If you are not sure if a list item applies to your research, read the appropriate section before selecting a response.

Materials & experimental systems

n/a	Involved in the study	
☐	☒	Antibodies
☐	☒	Eukaryotic cell lines
☒	☐	Palaeontology
☐	☒	Animals and other organisms
☐	☒	Human research participants
☐	☒	Clinical data

Methods

n/a	Involved in the study	
☐	☒	ChIP-seq
☐	☒	Flow cytometry
☒	☐	MRI-based neuroimaging

Antibodies

Antibodies used	AF-700 anti-mouse Ly6-G/Ly6C (Gr-1) (108422, Biolegend) - FACS<br>PE anti-mouse CD86 (105008, Biolegend) - FACS<br>APC-Cy7 anti-mouse CD117 (105826, Biolegend) - FACS<br>anti-mouse PU.1/SPI1 (sc325, Santa Cruz Biotechnology) - ChIP-seq, WB<br>anti-mouse KDM1/LSD1 (ab17721, Abcam) - WB<br>anti-mouse HSP60 (sc13966, Santa Cruz Biotechnology) - WB<br>anti-mouse H3K27ac (ab4729, Abcam) - ChIP-seq<br>anti-mouse H3K4me1 (ab8895, Abcam) - ChIP-seq<br>anti-mouse H3K4me2 (ab32356, Abcam) - ChIP-seq<br>anti-mouse H3K4me3 (ab8580, Abcam) - ChIP-seq<br>anti-mouse Med1/CRSP1/Trap220 (A300-793A, Bethyl Labs) - ChIP-seq<br>anti-mouse Brd4 (A301-985A100, Bethyl Labs) - ChIP-seq
Validation	All of the antibodies used have been extensively utilized in the literature and have been validated previously.

Eukaryotic cell lines

Policy information about cell lines

Cell line source(s)	Cell lines were either generated within the laboratory or were sourced from ATCC
Authentication	STR testing was performed to authenticate cell lines
Mycoplasma contamination	Cells were tested regularly for Mycoplasma infection, in line with the institute policy on Mycoplasma testing
Commonly misidentified lines (See ICLAC register)	No commonly misidentified cell lines were used.

Animals and other organisms

Policy information about studies involving animals; ARRIVE guidelines recommended for reporting animal research

Laboratory animals	C57BL/6 female mice<br>All mice were 6–8 weeks old<br>Further details in Methods
Wild animals	This study did not involve wild animals.
Field-collected samples	This study did not involve samples collected from the field.
Ethics oversight	All studies were conducted in accordance with the GSK Policy on the Care, Welfare and Treatment of Laboratory Animals and were reviewed by the Institutional Animal Care and Use Committee at GSK or were conducted under the approval of the institutional animal ethics review board and were authorized by the Animal Experimentation Ethics Committee (AEEC), Peter MacCallum Cancer Centre.

Note that full information on the approval of the study protocol must also be provided in the manuscript.

Human research participants

Policy information about studies involving human research participants

Population characteristics	Meta-analysis was performed on previously generated human research patient data - information on these datasets can be obtained from the original manuscripts.
Recruitment	Information on these datasets can be obtained from the original manuscripts.
Ethics oversight	Information on these datasets can be obtained from the original manuscripts.

Note that full information on the approval of the study protocol must also be provided in the manuscript.

Clinical data

Policy information about clinical studies

All manuscripts should comply with the ICMJE guidelines for publication of clinical research and a completed CONSORT checklist must be included with all submissions.

Clinical trial registration	NCT01943851
Study protocol	Subject will be administered a 5 milligram (mg) starting dose of GSK525762, oral tablets, QD. Dose escalations will be performed in Part 1 and dose adjustments are allowed to address tolerability and safety issues. Thereafter, subjects will be enrolled in a standard 3+3 design. Separate dose escalation cohorts will be opened for subjects with AML, NHL, and MM for QD dosing. Dose escalation will continue until an MTD is determined or until a dose of 200 mg per day is reached.
Data collection	BM-MNCs were separated using Ficoll density gradient. BM-MNCs were then cryopreserved at -80°C in 90% FBS and 10% DMSO. BM-MNCs samples from baseline, remission and relapse time points from BET001 and baseline and relapse from BET002 were rapidly thawed in a 37°C water bath. Cells were then transferred to a 50ml falcon tube and warm IMDM + 20% FBS + DNase1 (0.1mg/mL) culture medium was added drop wise to the cells. Cells were then washed with PBS + 1% BSA and subsequently processed for flow cytometry.<br><br>Patient samples were collected on site at presentation, during remission and at relapse. The timeline of collection, treatments and response are shown in Figure 1.
Outcomes	Patient response was assessed by BM-Blast percentage by a certified haematologist, as per standard clinical practice at the Peter MacCullum Cancer Centre.

ChIP-seq

Data deposition

☒ Confirm that both raw and final processed data have been deposited in a public database such as GEO.

☒ Confirm that you have deposited or provided access to graph files (e.g. BED files) for the called peaks.

Data access links<br>*May remain private before publication.*	The GEO accession number is GSE110901.
Files in database submission	fastq files<br>bed files
Genome browser session<br>(e.g. UCSC)	No longer applicable

Methodology

Replicates	All ChIP-seq experiments were performed with a single replicate, as is the standard in the field. ChIP-seq for all comparisons between experimental conditions was performed in a single experiment, in order to ensure fair and robust comparison between datasets. Some ChIP-seq datasets/experimental conditions were replicated and the data was concordant with the presented data.
Sequencing depth	Single-end 75bp reads. All samples were sequenced to a depth of at least ~10 million reads
Antibodies	anti-mouse PU.1/SPI1 (sc325, Santa Cruz Biotechnology)<br>anti-mouse H3K27ac (ab4729, Abcam)<br>anti-mouse H3K4me1 (ab8895, Abcam)<br>anti-mouse H3K4me2 (ab32356, Abcam)<br>anti-mouse H3K4me3 (ab8580, Abcam)<br>anti-mouse Med1/CRSP1/Trap220 (A300-793A, Bethyl Labs)<br>anti-mouse Brd4 (A301-985A100, Bethyl Labs)
Peak calling parameters	Peak calling was performed with MACS235 with default parameters. ChIP–seq coverage across selected genomic regions was calculated with BEDtools37.
Data quality	Reads were aligned to the mouse genome (GRCm38.78) with BWA-mem34.<br>Duplicate reads and reads mapping to blacklist regions or the mitochondria were removed. Quality was accessed by visual inspection and correlation with previously generated datasets.
Software	Sequencing was performed on the NextSeq500 and analysed using the standard analysis tools described in the Methods.

Flow Cytometry

Plots

Confirm that:

☒ The axis labels state the marker and fluorochrome used (e.g. CD4-FITC).

☒ The axis scales are clearly visible. Include numbers along axes only for bottom left plot of group (a 'group' is an analysis of identical markers).

☒ All plots are contour plots with outliers or pseudocolor plots.

☒ A numerical value for number of cells or percentage (with statistics) is provided.

Methodology

Sample preparation	Cells were obtained from culture, washed with PBS and re-suspended in PBS with appropriate concentration of antibody
Instrument	BD FACSAria III<br>BD FACSAria Fusion flow sorter<br>BD LSRFortessa X-20
Software	Collected in FACSDiva and analysed in FlowJo
Cell population abundance	Re-analysis was performed to ensure purity of the sorted populations

Gating strategy	Singlets were defined by FSC-A and FSC-H. Live cells were gated by morphology based on FSC-A and SSC-A. No positive or negative gates were used for flow analysis. For sorting, positive and negative gates were defined by using an unstained control and a negative control population.

☒ Tick this box to confirm that a figure exemplifying the gating strategy is provided in the Supplementary Information.
